# Successful Management of Infective Endocarditis Complicated by Pheochromocytoma: A Case Report

**DOI:** 10.7759/cureus.80269

**Published:** 2025-03-08

**Authors:** Koki Ikemoto, Akiyuki Takahashi, Kazunari Ohkawa, Keisuke Shuntoh, Katsuhiko Oka

**Affiliations:** 1 Department of Cardiovascular Surgery, Japanese Red Cross Society Kyoto Daiichi Hospital, Kyoto, JPN

**Keywords:** adrenalectomy, catecholamine, endocarditis, hypertension, pheochromocytomas

## Abstract

Pheochromocytomas present with paroxysmal hypertension due to a sudden release of catecholamines stimulated by radiological contrast media, surgery, or anesthetic agents. This often complicates the maintenance of patient hemodynamics during surgery. A 55-year-old man with a high fever was admitted to a hospital. Laboratory blood tests revealed elevated white blood cell and C-reactive protein levels. Transthoracic and transesophageal echocardiography revealed moderate aortic regurgitation, along with aortic valve vegetation. Magnetic resonance imaging revealed multiple cerebral embolisms, whereas computed tomography showed a left adrenal incidentaloma. Further examinations showed high levels of plasma-free metanephrine, adrenaline, and noradrenaline in the blood and metanephrines in the urine. 123I-metaiodobenzylguanidine scintigraphy revealed ligand accumulation in the tumor at 6 and 24 h after injection. Based on these results, the diagnosis of pheochromocytoma was confirmed. Doxazosin was promptly administered, and its dosage was escalated. Despite ongoing antimicrobial therapy, transesophageal echocardiography did not reveal any reduction in the size of the vegetation. Hence, the patient underwent surgical treatment. A laparoscopic left adrenalectomy was initially performed. The patient’s blood pressure increased with insufflation and manipulation around the tumor but dropped immediately after the adrenal tumor was resected. Following the adrenalectomy, a cardiopulmonary bypass was established. The bicuspid aortic valve leaflets along with the vegetation were completely resected. Subsequently, a mechanical aortic valve was implanted. Inotropic agents were completely weaned off within two days after surgery. A pathological examination confirmed the adrenal incidentaloma to be pheochromocytoma. One-stage surgery with adrenalectomy before cardiac surgery using cardiopulmonary bypass may be an effective strategy for patients with pheochromocytomas diagnosed with infective endocarditis. In addition, it can reduce the risk of complications with pheochromocytoma by managing the patient's systemic condition as much as possible before cardiac surgery.

## Introduction

Pheochromocytomas and paragangliomas (PPGLs) are rare neuroendocrine tumors originating from chromaffin cells in the adrenal medulla or extra-adrenal paraganglia that can synthesize, store, and secrete catecholamines [1]. The typical symptom triad includes headache, palpitations, and tachycardia [2], and the most common clinical manifestation is hypertension, occurring in approximately 80-90% of patients with PPGL. Approximately half of these patients develop sustained hypertension, whereas the other patients present with paroxysmal hypertension. Episodes of paroxysmal hypertension are often triggered by sudden catecholamine secretion, which can be induced by exercise, abdominal pressure, smoking, drugs like beta-blockades, surgery, anesthetic agents, radiologic contrasts, or tricyclic antidepressants [1,3-5]. They might contribute to blood pressure instability [6], which can be particularly challenging to manage as it is often resistant to antihypertensive therapy.

## Case presentation

A 55-year-old male farmer with a high fever and severe fatigue was initially admitted to a hospital. The body mass index of the patient was 18.3 kg/m2. Laboratory blood tests revealed elevated white blood cell counts, C-reactive protein levels, and uncontrolled diabetes mellitus (Table 1).

**Table 1:** Preoperative laboratory blood and urinary test

Test	Assay	Value	Normal range
Blood	Sodium (mEq/L)	158	138-146
	Potassium (mEq/L)	2.8	3.6-4.9
	Chlorine (mEq/L)	106	99-109
	C-reactive protein (mg/dL)	19.09	≦0.30
	White blood cells (×10^3^/μL)	10.24	4.00-8.00
	Hemoglobin A1c (%)	9.7	4.6-6.2
	plasma-free metanephrine (pg/mL)	294	<130
	plasma adrenaline (pg/mL)	2544	<100
	plasma noradrenaline (pg/mL)	4735	100-450
	plasma dopamin (pg/mL)	155	<20
Urinary	Adrenaline (μg/day)	353	3.4-26.9
	noradrenaline (μg/day)	666	48.6-168.4
	dopamin (μg/day)	219.8	365.0-961.5
	metanephrine (μg/day)	1.22	0.04-0.19
	normetanephrine (μg/day)	0.65	0.09-0.33

**Figure 1:**
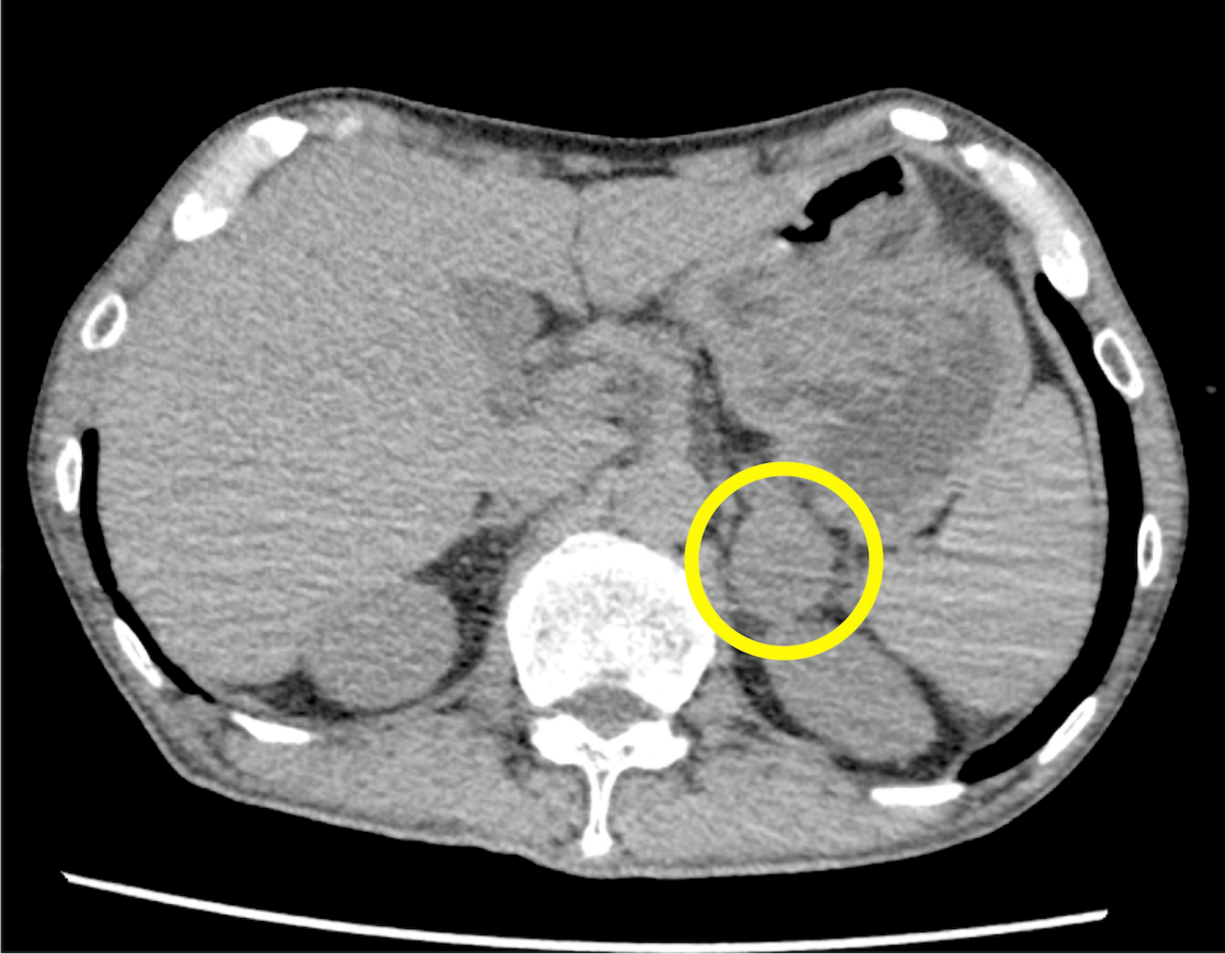
Whole-body computed tomography A left adrenal incidentaloma is observed (yellow circle).

Electrocardiography revealed rapid atrial fibrillation. Computed tomography detected a left adrenal incidentaloma with a diameter of 3 cm (Figure 1).

**Figure 2:**
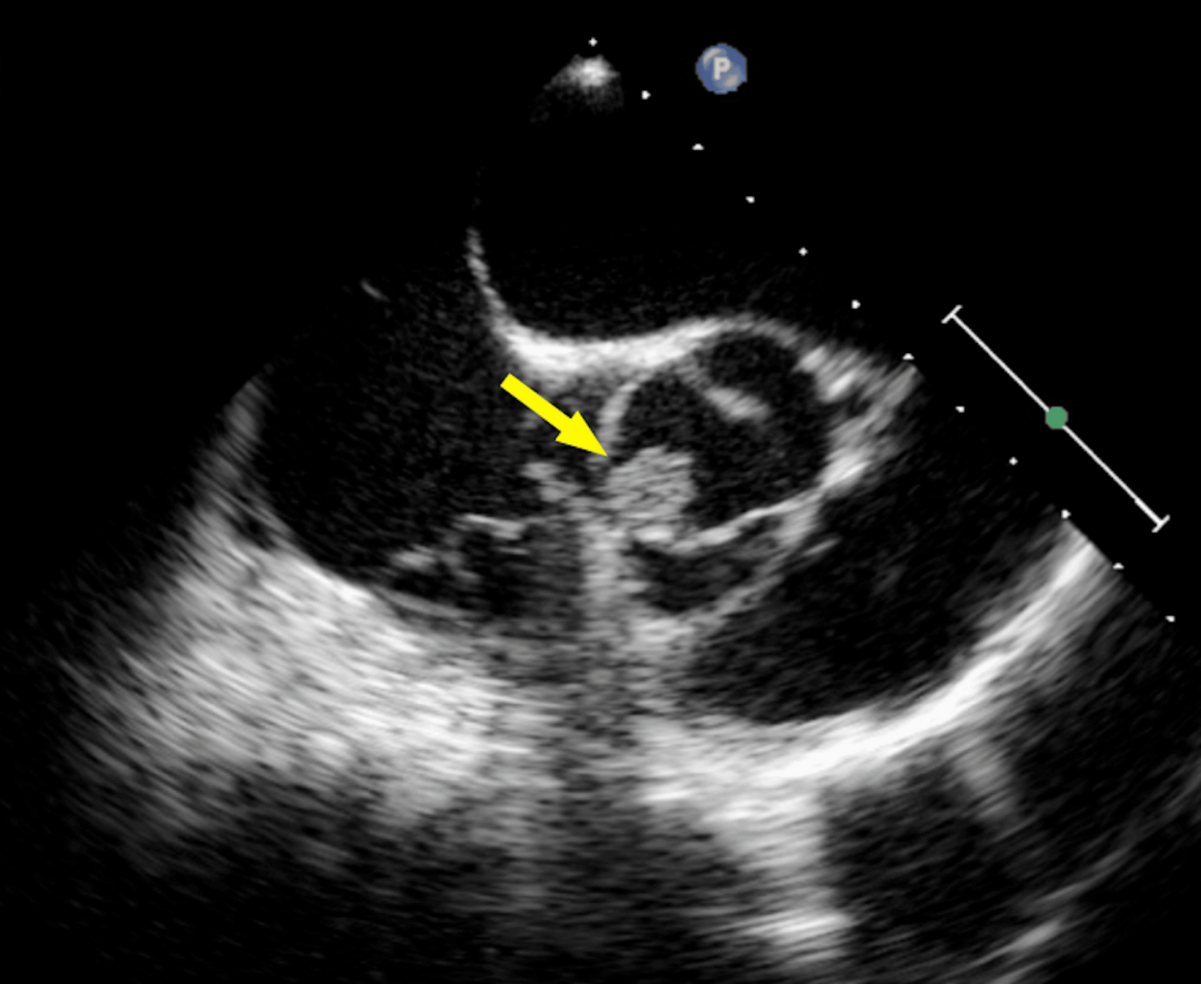
Transesophageal echocardiography at the initial hospital Vegetation is observed at the bicuspid aortic valve (yellow arrow).

Transthoracic echocardiography revealed moderate aortic regurgitation with a left ventricular ejection fraction of 50%, whereas transesophageal echocardiography showed a 12-mm vegetation on the aortic valve (Figure 2).

**Figure 3:**
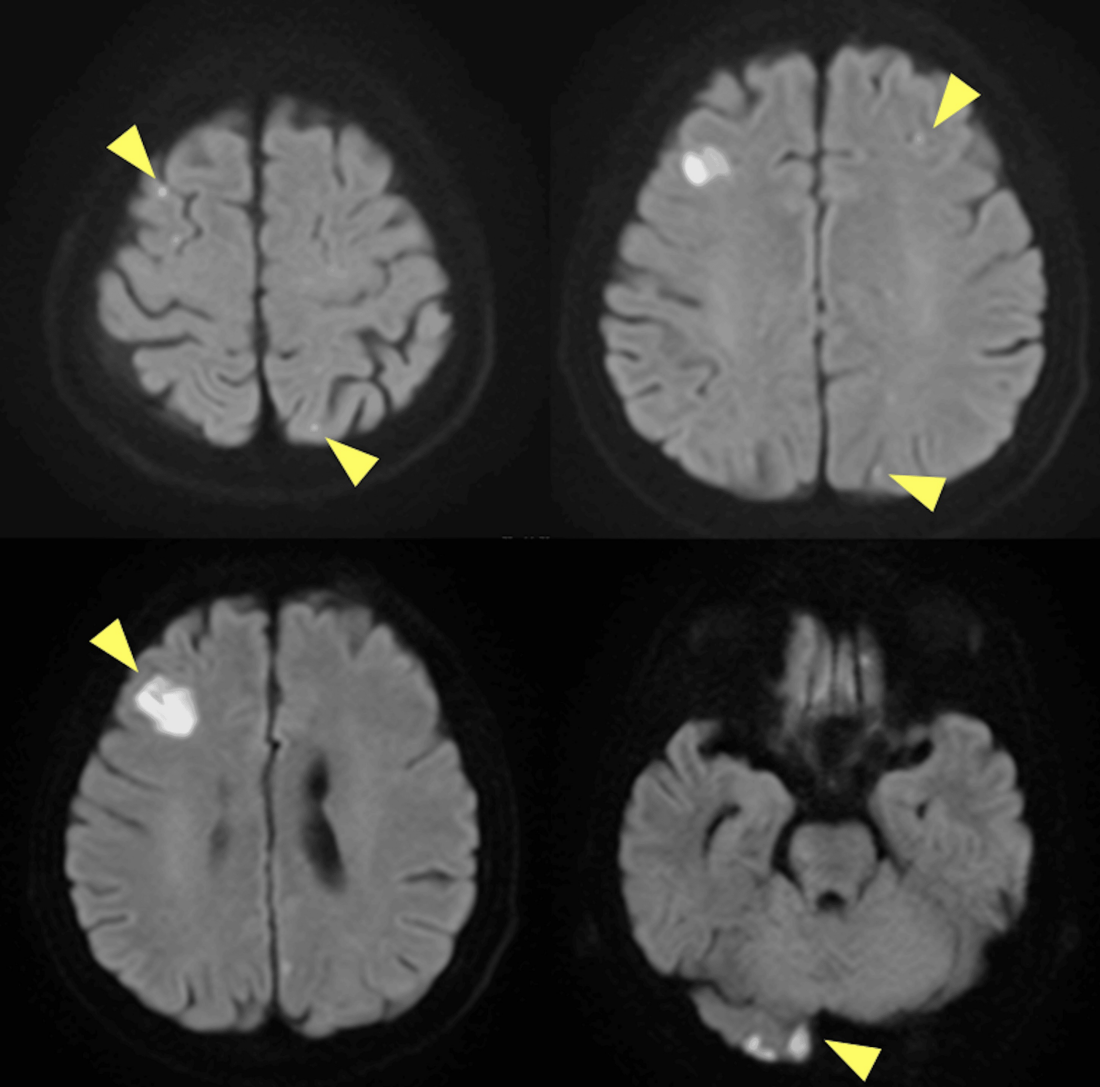
Magnetic resonance imaging of the whole brain There are multiple acute cerebral infarctions throughout the whole brain (yellow arrows).

Magnetic resonance imaging revealed multiple cerebral embolisms (Figure 3).

Piperacillin/tazobactam administration was immediately initiated following the diagnosis of infective endocarditis, and it was altered two days later to cefazolin targeting *Staphylococcus aureus* detected by blood culture examination. Further investigation of the left adrenal incidentaloma showed high levels of plasma and urinary catecholamines. Additional tests revealed a high level of plasma-free metanephrine. Hypernatremia and hypokalemia were also observed (Table 1).

**Figure 4:**
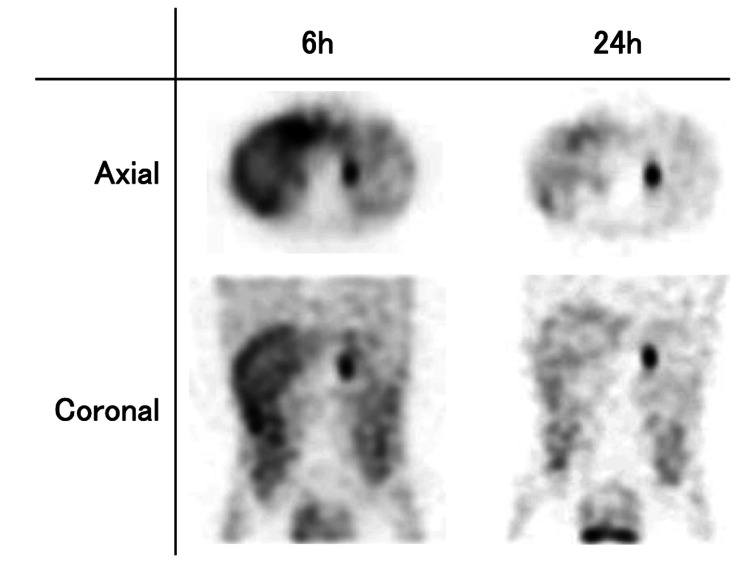
123I-metaiodobenzylguanidine scintigraphy showing axial and coronal views The ligand accumulates in the tumor at 6 and 24 h after injection.

123I-metaiodobenzylguanidine scintigraphy detected ligand accumulation in the tumor at 6 and 24 h after injection (Figure 4).

Based on these results, the diagnosis of pheochromocytoma was confirmed. Doxazosin was promptly administered, followed by bisoprolol, and was gradually increased to 12 mg/day. Potassium-supplemented 5% glucose solution (1500 mL/day) administration was continued to correct the electrolyte imbalance and stabilize the circulating blood volume. Transesophageal echocardiography performed a week after the referral did not reveal any reduction in the size of the vegetation (Videos 1, 2). Therefore, the patient underwent surgical treatment.

**Video 1:** Transesophageal echocardiography at our hospital (long-axis view)

**Video 2:** Transesophageal echocardiography at our hospital (short-axis view) The vegetation did not shrink despite appropriate antibiotic therapy.

**Figure 5:**
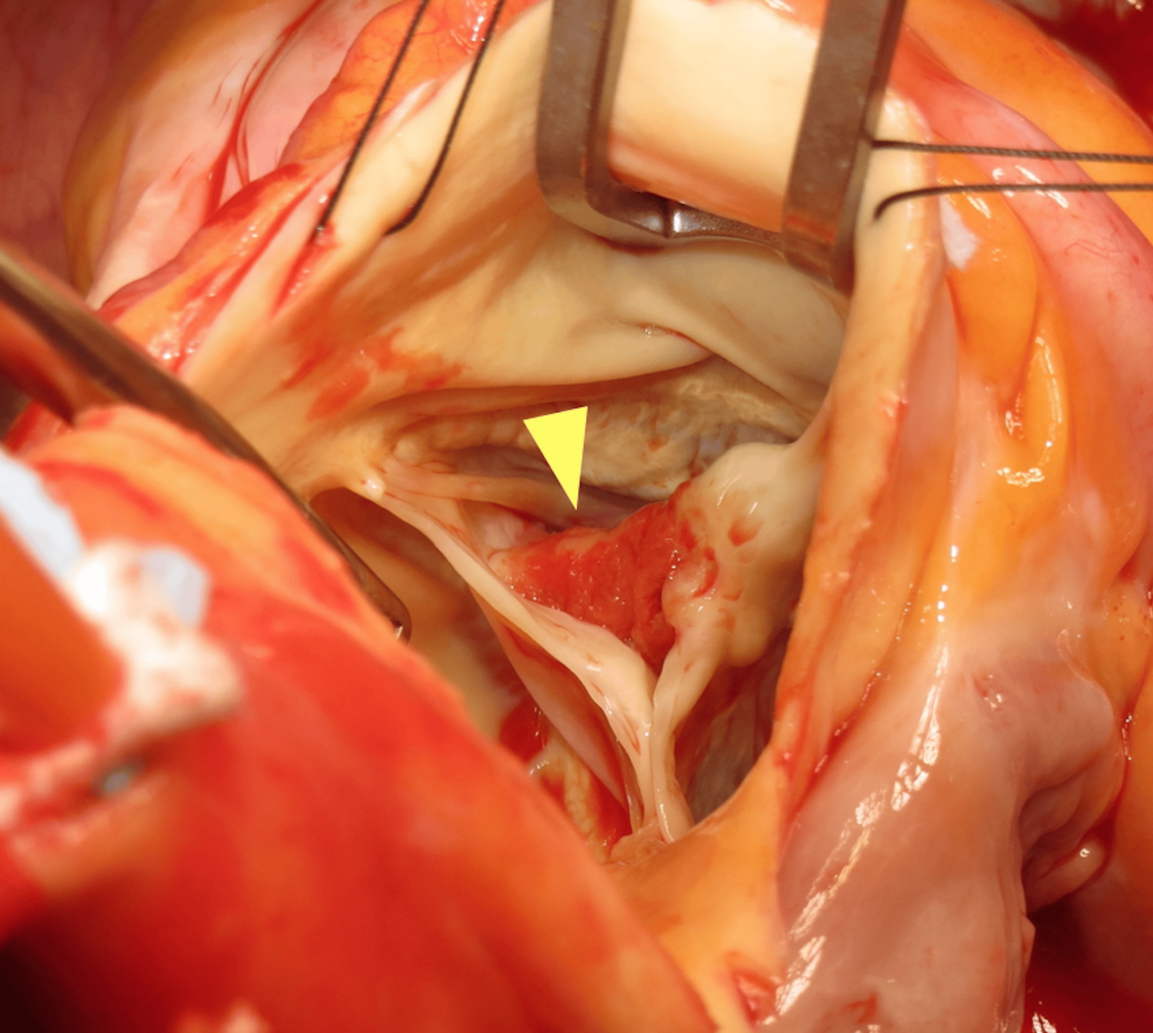
Surgical image of the aortic valve Vegetation is attached to the raphe between the right and non-coronary cusps (yellow arrow).

Left adrenalectomy was performed through a laparoscopic transperitoneal approach in the right lateral position, as making a large incision during open surgery to remove the adrenal tumor could facilitate the secretion of catecholamines. Mean arterial pressure (MAP) and heart rate were stabilized during anesthesia induction using sevoflurane, remifentanil, and rocuronium. The MAP increased during insufflation and manipulation around the tumor, and it was managed using nitroglycerin and nicardipine. However, the MAP dropped immediately after the adrenal tumor was carefully resected. Therefore, dobutamine and noradrenaline were administered at their initial doses to stabilize the patient’s hemodynamics. Following adrenalectomy, cardiopulmonary bypass (CPB) was established with a median sternotomy in the supine position. An aortotomy was performed on the ascending aorta after cardiac arrest. The bicuspid aortic valve, with fused right and non-coronary cusps, was inspected (Figure 5). The aortic valve leaflets were resected, and the vegetation was carefully curetted. Subsequently, a St. Jude Medical Regent (Abbott, Chicago, USA) mechanical aortic valve measuring 25 mm was inserted using 13 horizontal mattress sutures.

**Figure 6:**
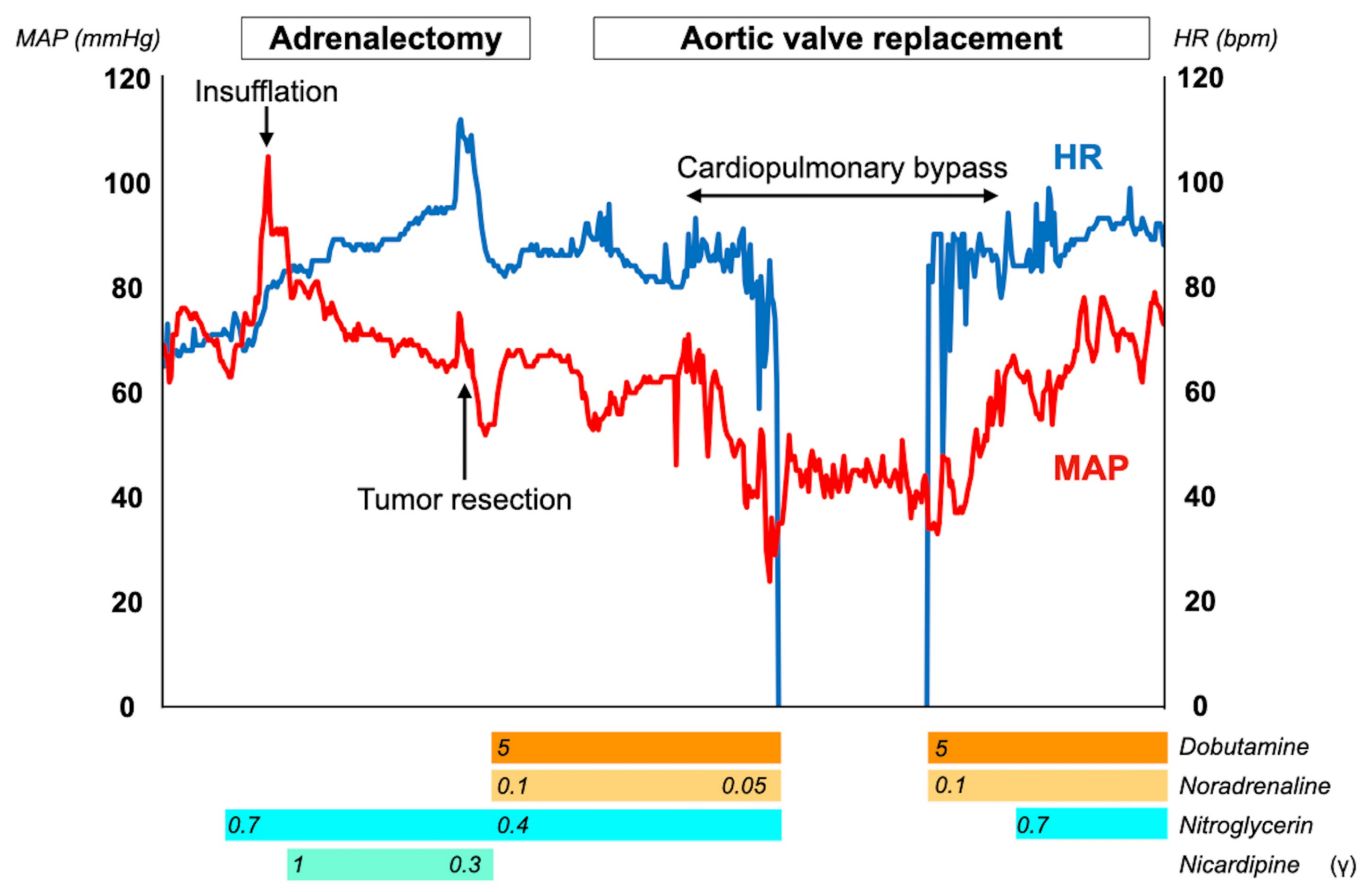
Transition of the vital signs during surgeries The MAP initially increased after insufflation, and the MAP and HR increased during tumor manipulation. However, they promptly dropped after tumor resection. The patient’s condition was well-controlled before and after cardiac surgery with cardiopulmonary bypass. MAP: mean arterial pressure, HR: heart rate.

CPB was safely weaned off with an initial dose of dobutamine and noradrenaline (Figure 6).

**Figure 7:**
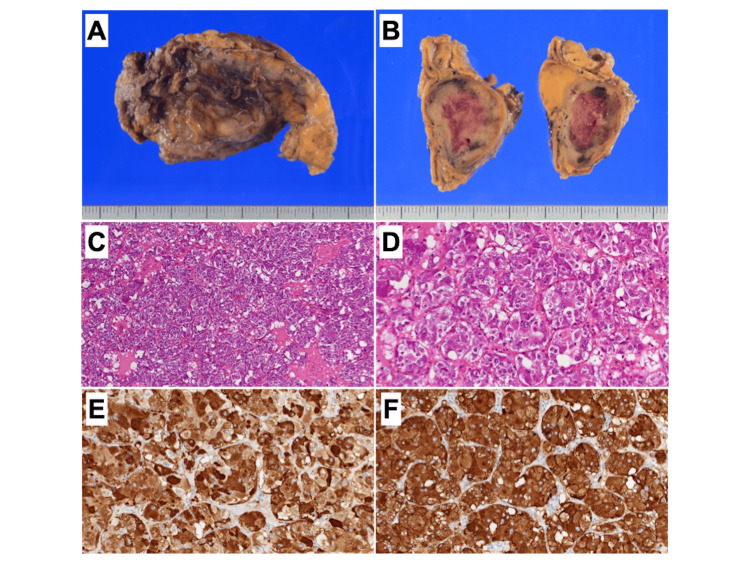
Pathological examination of the excised left adrenal tumor A solid tumor with clear borders is observed in the adrenal gland, and macroscopic bleeding is detected within the tumor (A, B). Hematoxylin and eosin staining showing cell nests of different sizes, consisting of large tumor cells in an irregular zellballen pattern (C, D). Chromogranin and synaptophysin are positive on immunohistochemical staining (E: chromogranin A, F: synaptophysin).

The postoperative course was uneventful, and the inotropic agents were completely weaned off within two days. A pathological examination confirmed the excised adrenal incidentaloma to be pheochromocytoma (Figure 7). 

After six weeks of antimicrobial therapy following the surgery, the patient was discharged from our institution on postoperative day 50 and continued to receive outpatient monitoring in cardiology and endocrinology clinics.

## Discussion

In our patient diagnosed with infective endocarditis complicated with pheochromocytoma, one-stage surgery with adrenalectomy followed by cardiovascular surgery using CPB was a successful strategy. Some studies have reported individual cases of staged procedures involving cardiovascular surgery and adrenalectomy [7,8]. Nevertheless, there are limited reports on combined cardiovascular surgery procedures using CPB and adrenalectomy. Moreover, in these cases, there was an adequate duration of preoperative medication, and adrenalectomy was performed after cardiac surgery [9,10]. In our case, there was insufficient time to elevate the doxazosin dose to the recommended level before surgery or to sufficiently control diabetes mellitus. In addition, an adrenalectomy was performed before cardiac surgery. One reason for this was the urgency of surgical intervention due to multiple cerebral infarctions and persistent vegetation. Another reason was that the initiation and withdrawal of CPB could trigger a catecholaminergic crisis, posing challenges to hemodynamic control under the existence of pheochromocytomas. Despite these complexities, the patient underwent aortic valve replacement and adrenalectomy without encountering any complications. Therefore, we believe that one-stage surgery with adrenalectomy before cardiovascular surgery using CPB has significant benefits, particularly when there is insufficient time for preoperative medication.

Preoperative management of pheochromocytomas is essential, even if it may not be adequate. Emergency surgery is often recommended for infective endocarditis that leads to heart failure, sepsis, and repeated cerebral embolization. Vegetation that is unresponsive to antimicrobial therapy also requires early intervention [11]. In our report, urgent surgery was considered necessary because the vegetation had provoked multiple cerebral infarctions. However, we decided to proceed with the management of the pheochromocytoma, considering the potential surgical complications in untreated cases. Although urgent surgery is often unavoidable depending on the form of vegetation and systemic status, preoperative management for pheochromocytoma should be considered when it offers greater benefits.

## Conclusions

One-stage surgery with adrenalectomy before cardiovascular surgery using CPB may be an effective strategy in patients who need urgent surgical intervention with infective endocarditis complicated with pheochromocytomas. This approach does not necessitate systemic management between surgeries and can facilitate hemodynamic control during cardiac surgery as the pheochromocytoma has already been removed. Additionally, the preoperative management of pheochromocytomas with medication, volume adjustment, and electrolyte correction is crucial, as patients may experience some problems when pheochromocytomas are first diagnosed.
